# Supplementary material for: Exploring the Impact of a Vicarious Learning Approach on Student Pharmacists’ Professional Identity Formation Using a Simulated Pharmacist–Patient Encounter
Source: Pharmacy (Basel). 2023 Nov 16;11(6):177. doi: 10.3390/pharmacy11060177 (PMC10661241; doi:10.3390/pharmacy11060177)
Supplement: Supplementary file 1 [file pharmacy-11-00177-s001.zip › PIF Script_final .pdf]

1. Scenario:
- Pharmacy practice exam room
- Patient presenting for DM injectable teaching and medication review
    - o Metformin bottle
    - o Statin bottle
    - o New start – injection

Courtney: Hello, my name is Courtney. I am the pharmacist that works with your doctor. How are you doing today?

Kate: Good. How are you?

Courtney: Good. Your doctor wanted us to meet to review your medications and recent lab work. It will probably take 5-10 minutes. Would that be okay with you?

Kate: Yes.

Courtney: Perfect. Before we get started can I just verify your name and DOB?

Kate: Yes, my name is …. and my DOB is ….

Courtney: Great. I see you brought in your medication bottles. That is very helpful so I can see exactly what you are taking at home. Do you mind if I take a look?

Kate: Of course. *hands over bottles (metformin and atorvastatin)*

Courtney: *Reviews name, dose, route, frequency, indication in depth. Will open bottles and show medication as discussing*

Kate: *Nods in agreement while reviewing medications. Answers any questions regarding how medications are taken. Confirms compliance*

Courtney: Great. Well it sounds like you have good knowledge of the medications you are currently taking and have a good system in place in order for you to take them consistently as prescribed! Now taking a look at your recent blood work. Your A1c, which is the average of your blood sugars over a 3 month period, came back at 8.6%. This number is a little high as we typically target an A1c less than 7%.

Kate: Oh no, that is much higher than I remember it being the last time I got it checked.

Courtney: Yeah, there is definitely some room for improvement. It is important that we get your sugars back down because if they stay too high for too long it can cause damage to the nerves, eyes, and kidneys.

Kate: Yeah, I definitely want to work on getting my sugars back down.

Courtney: I think the best option for you would be to add on a new medication, specifically a Glucagon-Like Peptide-1 Receptor Agonist. These medications work to increase glucose-dependent insulin secretion and decrease inappropriate glucagon secretion. It also slows gastric emptying and acts in areas of the brain to help regulate appetite and caloric intake which can lead to weight loss benefits in addition to lowering blood glucose. Another benefit with this class of medications is they have shown to reduce risk of major cardiovascular events. As far as side effects, the most common one to expect is

gastrointestinal upset. Do you know if you or your family have any history of medullary thyroid carcinoma or multiple endocrine neoplasia syndrome type 2? And do you have any personal history of gastroparesis or pancreatitis?

Kate: *seems confused* Oh um I don't think so? What kind of medication is it again? And I would have to take it in addition to my other medications? I already take the metformin for my diabetes.

Courtney: Good because we would want to avoid this medication with those conditions. The name of the medication I would like to start you on is called Ozempic which is an injection you would have to give yourself once a week. And yes, you would be taking Ozempic in addition to your metformin.

Kate: *seems apprehensive* Oh, I'm not sure I like the thought of having to take another medication and I definitely don't love the thought of using an injection…

Courtney: I really think this is the best option for you in order to get your sugars back to goal and the needle is tiny. Here let me show you! *Thoroughly walk through administration of Ozempic. Will discuss dosing, titration, storage, disposal, rotating injection sites, etc.*

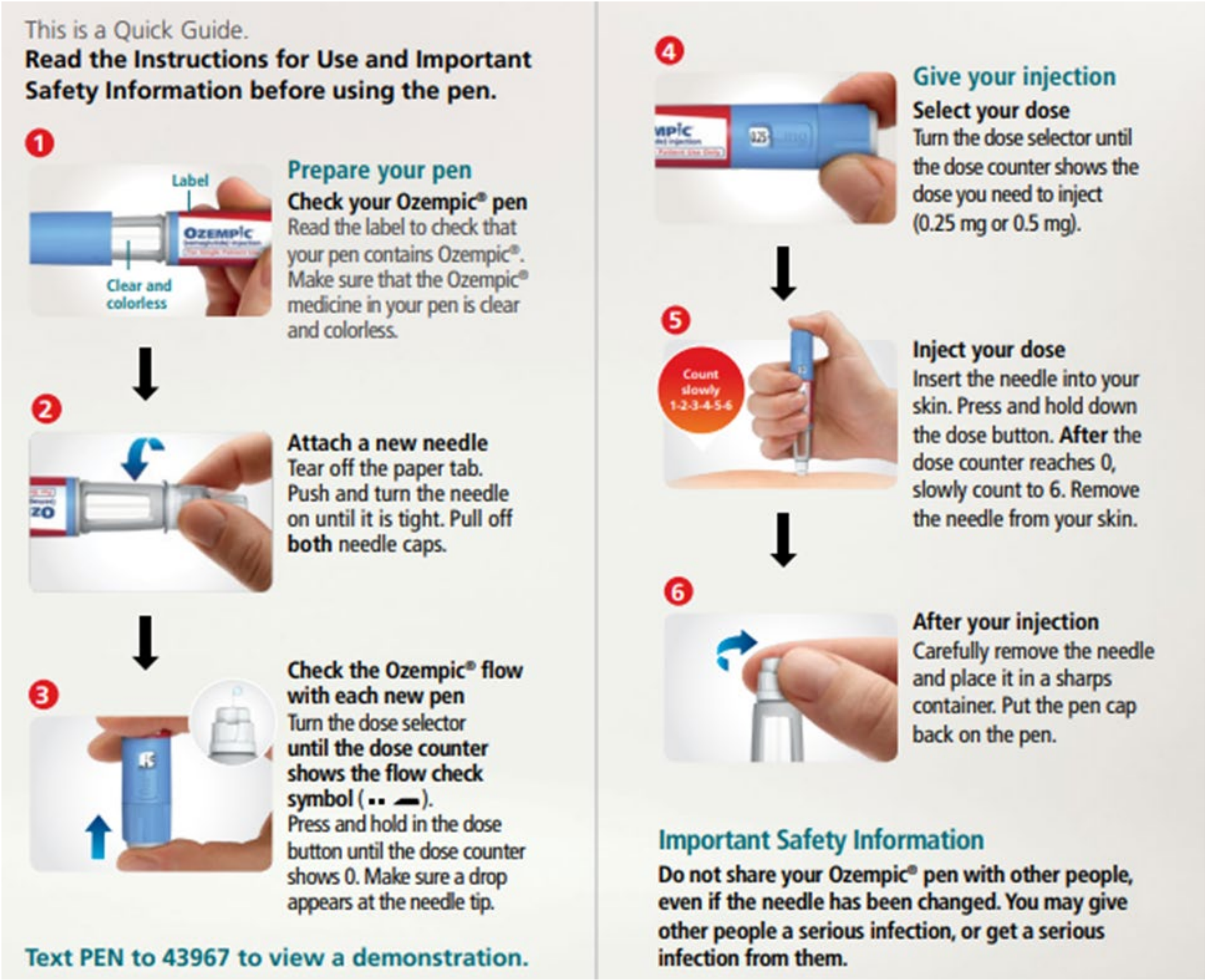

Kate: *feeling a little more comfortable* That doesn't seem too bad.

Courtney: Good! Why don’t you give it a try to make sure you are comfortable with the pen before your first dose.

Kate: *Repeats administration steps with demo pen*

Courtney: You seem ready to start this medication right away!

Kate: Yeah, I guess I could handle the injection especially since it’s just once a week. Do you know if this will be covered by my insurance? I have insurance through my employer, but my budget has been pretty tight lately.

Courtney: This medication can be pretty expensive, but it is typically covered by most insurances. I will follow up on the prescription once it is sent to the pharmacy to ensure it is covered. If it needs any additional authorization through your insurance I will make sure to complete the forms right away. Ozempic also has a savings card available online. This coupon can help reduce your copay to as low as $25 for up to 2 years.

Kate: I would be able to afford that! I appreciate you checking on the coverage of my prescription and will definitely use the coupon. Thank you so much!

Courtney: Of course! I know we talked about a lot today. Do you have any remaining questions?

Kate: I don’t think so... Well actually, I’m still not sure about the side effects and the family history questions you mentioned earlier. Could we review that again?

Courtney: Sure! Here is the package insert which discusses everything we had talked about and more. If any other questions come up after reviewing this information or once you start the medication you can give the office a call and they will get you in touch with me. Otherwise, I will plan to call you in 1-2 weeks to see how your first couple of doses went.

Kate: Okay (apprehension)... I guess that sounds like a plan (assurance). I do appreciate your help. I will pick the medication up from the pharmacy and try to start it today.

Courtney: Great! Have a good rest of your day.
