# Supplementary material for: Exploring the Impact of a Vicarious Learning Approach on Student Pharmacists’ Professional Identity Formation Using a Simulated Pharmacist–Patient Encounter
Source: Pharmacy (Basel). 2023 Nov 16;11(6):177. doi: 10.3390/pharmacy11060177 (PMC10661241; doi:10.3390/pharmacy11060177)
Supplement: Supplementary file 1 [file pharmacy-11-00177-s001.zip › PIF Exercise & Critical Reflection -Assignment and Rubric 2023.pdf]

PIF Exercise & Critical Reflection: Assignment and Grading Rubric

2023 IPS6 (IPS 502)

Within the pharmacy curriculum, students have been provided several opportunities to experience personal & professional growth along their journey to becoming a pharmacist, with the over-arching goal to engage students in their Professional Identity Formation (PIF). PIF is an evolutionary process in which student pharmacists begin to **think, act, and feel** like a pharmacist over the passage of time, and begin to gain a true sense of belonging to the pharmacy community that they are joining. This PIF exercise was developed for the capstone course Advanced IPS as a method for you to demonstrate your PIF to date in terms of how you might think, act, and feel like a pharmacist when reacting to a Pharmacist-Patient Encounter (PPE). Keep in mind that as a pharmacist, you may evaluate a patient's pharmacotherapy regimen, and tweak the medications/dosages perfectly for the individual patient's goals, but if the patient doesn't take the medication regimen correctly or at all, then perhaps all your hard work on optimizing the medication regimen was for naught. A sampling of **foundational pharmacy domains** that could influence a patient's adherence with a prescribed regimen, and which you will observe and reflect upon in this PPE, include:

- **Medication Safety/Patient Safety** principles (5 "Rights": patient, drug, time, dose, route)
- **Health Literacy** principles (what grade level is appropriate for the general population?)
- **Social Determinants of Health** (what barriers exist that could preclude the patient from adhering?)
- **Motivational Interviewing/Empathic Communication** strategies (building a trustworthy patient-provider relationship; empowering the patient to have a voice in their medication regimen)

The Pharmacist-Patient Encounter Exercise: You will view a 12-minute simulated Pharmacist-Patient Encounter in which the Pharmacist incorporates components of the four foundational pharmacy domains (above) into the dialogue, and you will then complete the following exercise.

1. Choose one (1) foundational topic domain (above) and write two sentences as to **what the pharmacist did well** (within the chosen domain); include your **specific justification** for why/how the pharmacist did a good job in that domain.
2. Choose one (1) foundational topic domain (above) and write two sentences as to **what the pharmacist did not perform optimally (**within the chosen domain); include your **specific justification** for why/how the pharmacist did not perform as well within that domain.
3. Complete a critical reflection, per instruction below.

Critical Reflection Assignment: PIF in Capstone Course: Think, Act & Feel Like a Pharmacist:

In 350-400 words, critically reflect upon your viewing of this PPE. As a student pharmacist, what is the impact of this PPE upon you/your learning, either positively or negatively? As a result of reflecting

upon this pharmacist's component of the encounter (and its apparent impact on the patient), what are you now motivated to do (or do differently) in your (immediate or longer term) future when you encounter patients?

Rubric: Maximum point value = 8

Assessment	4 (Critical Reflection)	3 (Reflection)	2 (Understanding)	1 (Non-reflection)	0 (Not acceptable)
Critical Reflection	Student provides sufficient description and critical analysis of the pharmacist-patient encounter **and applies it to future practice/behaviors.** Reflection is thorough, thoughtful, detailed, and clear.	Student provides sufficient description and analysis of the pharmacist-patient encounter but does not apply it to future practice/behaviors.	Student provides superficial summary or is lacking description and analysis of personal impact of the pharmacist-patient encounter's message.	Reflection on the impact of viewing the pharmacist-patient encounter is incomplete or unclear.	No reflection is submitted.
Foundational Topic Domain -performed well			1 Domain selected and justification for why it was performed well is included.	1 Domain selected, but not well-justified.	Domain not selected and justified.
Foundational Topic Domain -not performed well			1 Domain selected and justification for why it was not performed well is included.	1 Domain selected, but not well-justified.	Domain not selected and justified.

April 10, 2023 LLB
